# Loss of PIKfyve Causes Transdifferentiation of *Dictyostelium* Spores Into Basal Disc Cells

**DOI:** 10.3389/fcell.2021.692473

**Published:** 2021-08-19

**Authors:** Yoko Yamada, Gillian Forbes, Qingyou Du, Takefumi Kawata, Pauline Schaap

**Affiliations:** ^1^School of Life Sciences, University of Dundee, Dundee, United Kingdom; ^2^Department of Biology, Faculty of Science, Toho University, Funabashi, Japan; ^3^Department of Materials and Life Sciences, Faculty of Science and Technology, Sophia University, Tokyo, Japan

**Keywords:** 1-phosphatidylinositol-3-phosphate 5-kinase, Atg18, Vac14, membrane scission, vesicle trafficking, cell-type specific gene expression, sporulation, *Dictyostelium*

## Abstract

The 1-phosphatidylinositol-3-phosphate 5-kinase PIKfyve generates PtdIns3,5P2 on late phagolysosomes, which by recruiting the scission protein Atg18, results in their fragmentation in the normal course of endosome processing. Loss of PIKfyve function causes cellular hypervacuolization in eukaryotes and organ failure in humans. We identified *pikfyve* as the defective gene in a *Dictyostelium* mutant that failed to form spores. The amoebas normally differentiated into prespore cells and initiated spore coat protein synthesis in Golgi-derived prespore vesicles. However, instead of exocytosing, the prespore vesicles fused into the single vacuole that typifies the stalk and basal disc cells that support the spores. This process was accompanied by stalk wall biosynthesis, loss of spore gene expression and overexpression of *ecmB*, a basal disc and stalk-specific gene, but not of the stalk-specific genes *DDB_G0278745* and *DDB_G0277757*. Transdifferentiation of prespore into stalk-like cells was previously observed in mutants that lack early autophagy genes, like *atg5, atg7, and atg9*. However, while autophagy mutants specifically lacked cAMP induction of prespore gene expression, *pikfyve*^−^ showed normal early autophagy and prespore induction, but increased *in vitro* induction of *ecmB*. Combined, the data suggest that the *Dictyostelium* endosomal system influences cell fate by acting on cell type specific gene expression.

## Introduction

Differential phosphorylation of the inositol moiety of phosphatidylinositol (PtdIns) membrane lipids regulates a broad range of cellular processes. Position-specific PtdIns kinases and phosphatases determine the phosphorylated state and are themselves under stringent regulation. PIP3K5 (1-phosphatidylinositol-3-phosphate 5-kinase), also known as PIKfyve or Fab1, phosphorylates PtdIns3P on the inositol-5 position, and was first recognized as a major regulator of vacuole size in yeast, where Fab1 is activated by hyperosmotic stress (Gary et al., 1998). Yeast Fab1 acts in a complex with the scaffolding protein Vac14, the PtdIns 5-phosphatase Fig4, the autophagy protein Atg18 and the transmembrane protein Vac7 (McCartney et al., 2014). The mammalian ortholog PIKfyve and the other proteins, except Vac7, are also present in mammals, where loss of PIKfyve is embryonic lethal, while full or partial loss of Vac14 or Fig4 function result in massive spongiform defects in brain and heart as well as extensive vacuolation of other tissues (see Hasegawa et al., 2017).

In yeast, the complex of Fab1, Fig4, and Vac14 proteins localizes to the vacuole, while the mammalian orthologs localize to endosomes and lysosomes. This recruitment is achieved by binding of the FYVE domain of Fab1/PIKfyve to PtdIns3P in the membranes of these organelles. Vac14 acts as a scaffold with separate binding sites for PIKfyve and Fig4 (see Ho et al., 2012). Atg18, which has an additional role in early autophagosome formation (Grimmel et al., 2015), has different lipid binding sites for PtdIns3P and PtdIns3,5P2. Its interaction with PtdIns3,5P2, generated by PIKfyve activity, causes the multimerization of Atg18 and activation of its membrane scission activity, which fragments vacuoles into smaller vesicles as part of their normal processing (Gopaldass et al., 2017).

We identified *pikfyve* as the defective gene in a *Dictyostelium discoideum* mutant with defective spore formation. *Dictyostelium* amoebas feed on bacteria, but aggregate to form multicellular migrating slugs, when starved. The slugs transform into fruiting bodies and differentiate into a mass of walled spores that is supported by a column of vacuolated walled stalk cells and a basal disc consisting of stalk-like cells. *D. discoideum* is a popular model for study of a range of cell biological processes, such as cell migration and vesicle trafficking. PIKfyve was found earlier to be required for delivery of V-ATPase and proteases to early phagosomes in *D. discoideum* and to effectively kill captured *Legionella* bacteria (Buckley et al., 2019). We here report that loss of *pikfyve* causes transdifferentiation of maturing spores into cells that are transcriptionally and phenotypically similar to basal disc cells.

## Materials and Methods

### Cell Culture and Development

*D. discoideum* Ax2 was cultured either in HL5 axenic medium (Formedium, United Kingdom) or on SM agar plates in association with *Klebsiella aerogenes*. For development, cells were distributed at 2.5 × 10^6^ cells/cm^2^ on non-nutrient agar (1.5% agar in 8.8 mM KH_2_PO_4_ and 2.7 mM Na_2_HPO_4_) and for β-galactosidase staining on dialysis membrane supported by non-nutrient agar. Visualization of β-galactosidase expression in intact structures was performed using established procedures (Dingermann et al., 1989).

### DNA Constructs

To disrupt *pikfyve*, two fragments, KO5′ and KO3′, were amplified from Ax2 genomic DNA using primer pairs pikfyve-f1/pikfyve-r1 and pikfyve-f2/pikfyve-r2, respectively. After subcloning into pJet1.2blunt, KO5′, and KO3′ were cloned into pLPBLP (Faix et al., 2004) using the *Sal*I/*Hin*dIII sites of both fragment and plasmid for KO5′ and the *Pst*I/*Bam*HI sites for KO3′. This yielded plasmid pPikfyveKO, in which the LoxP-Bsr cassette is flanked by KO5′ and KO3′. The plasmid was linearized with *Bam*HI and transformed into Ax2 cells. Genomic DNAs from blasticidin resistant clones were screened by two PCR reactions for homologous recombination events (Supplementary Figure 1).

To disrupt fig4, two fragments, KO5′ and KO3′, were amplified from Ax2 gDNA using primer pairs Fig4I5′/Fig4I3′ and Fig4II5′/Fig4II3′. Using the restriction sites that were introduced in the primers, fragments KO5′ and KO3′ were successively inserted into the *Kpn*I/*Sal*I and *Pst*I/*Spe*I digested vector pLPBLP to generate pFig4KO. The KO construct was excised with *Kpn*I and *Spe*I and introduced into Ax2. Blasticidin resistant clones were screened by PCR for homologous recombination (Supplementary Figure 7A).

To disrupt *vac14*, two fragments, KO5′ and KO3′ were amplified from Ax2 gDNA using primer pairs Vac14I5′/Vac14I3′ and Vac14II5′/Vac14II3′, respectively. Using the restriction sites that were introduced in the primers, fragments KO5′ and KO3′ were successively inserted into *Kpn*I/*Sal*I and *Nde*I/*Bam*HI digested vector pLPBLP to generate vector pVac14KO. The KO construct was excised with *Kpn*I and *Bam*HI and introduced into Ax2. Blasticidin resistant clones were screened by PCR for homologous recombination (Supplementary Figure 7B).

### Induction of Gene Expression and Cell Differentiation

Induction of stalk cell differentiation in monolayers was performed as previously described (Berks and Kay, 1988; Yamada and Schaap, 2019). For induction of gene expression, cells transformed with cell-type specific promoters fused to the *lacZ* reporter were developed to tipped mounds or loose aggregates for stalk or prespore gene induction, respectively, dissociated, and incubated as 90 μl aliquots in microtiter plates for several hours. Plates were frozen and cells lysed by freeze-thawing three times under vigorous shaking. B -galactosidase activity was assayed by adding 30 μl of 2.5 × Z buffer and 10 μl of 10 mg/ml ONPG (o-nitrophenyl β-D-galactopyranoside) or 40 mM CPRG (chlorophenolred-β-D-galacto pyranoside) to the cell lysates, and by measuring OD_420_ or OD_574_, respectively, for calculation of ΔOD/min (Schaap et al., 1993).

### Quantitation of Autophagy

Cells transformed with RFP-GFP-Atg8 (Kimura et al., 2007) were starved on non-nutrient agar at 4°C overnight and then at 22°C for 1–2 h until aggregates were forming. Cells were harvested with 20 mM K-phosphate buffer, pH 6.2 (KK2), placed in a glass-bottomed dish and overlayed with a ∼0.75 mm thick layer of 1% agarose in 8.8 mM KH_2_PO_4_ and 2.7 mM Na_2_HPO_4_. Z-series of images in 0.3–0.5 μm steps were captured using a Nikon A1R + confocal microscope. Maximum intensity projections of the images were used to quantify fluorescent vesicles (Yamada and Schaap, 2020).

## Results

### Identification of *Pikfyve* as the Defective Gene in a Sporulation-Deficient Mutant

To identify genes that control *D. discoideum* sporulation, Ax2 cells, transformed with a fusion construct of monomeric RFP and the spore coat gene *cotC*, were subjected to restriction enzyme mediated insertional (REMI) mutagenesis (Kuspa and Loomis, 1992; Yamada et al., 2018). The mutant population was screened for mutants with defective CotC-mRFP expression and/or missing or abnormal spores. A clone, RT11da2, was isolated with both reduced CotC-mRFP expression and abnormal fruiting body morphology.

**FIGURE 1:**
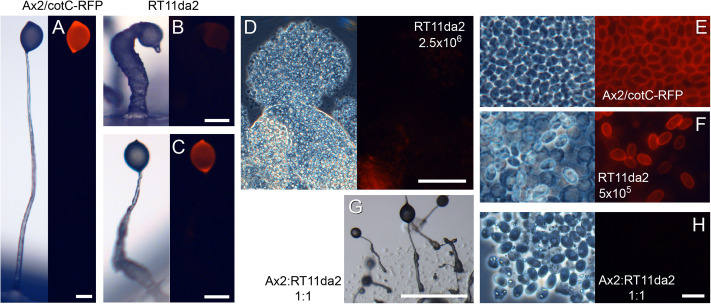
Phenotype of REMI clone RT11da2. Ax2/cotC-mRFP **(A,E)** and REMI clone RT11da2 **(B–D,F)** or a 1:1 mixture of RT11da2 and unlabeled Ax2 **(G,H)** were developed on non-nutrient agar at 2.5 × 10^6^ cells/cm^2^ **(A,B,D,G,H)** or 5 × 10^5^ cells/cm^2^ **(C,F)**. Terminal structures were photographed *in situ* under transmitted light **(A–C,G)** and epi-fluorescence **(A–C)** or under phase contrast and epifluorescence after being squashed under a coverslip **(D–F,H)**. **(E,F,H)** Show part of the spore head. Bars **(A–D)** 100 μm, **(E,F,H)** 10 μm, **(G)** 1 mm.

When plated at a standard cell density of 2.5 × 10^6^ cells/cm^2^, RT11da2 cells showed delayed formation of abnormal fruiting bodies (compare Figures 1A,B) and left many unaggregated cells behind on the agar. The cells inside the fruiting structures and the unaggregated cells were often highly vacuolated (Figure 1D) and no spores were observed. Plating at a 5–10-fold lower cell density improved fruiting body formation (Figure 1C), but lower stalks were still enlarged. Some spore heads contained normal spores with RFP-positive walls, like the Ax2/cotC-mRFP parent. However, in many structures, there were also spores with large vacuoles or vacuolated cells without CotC-mRFP fluorescence. Mixing RT11da2 cells with Ax2 cells also improved development, but the chimeric structures still showed enlarged lower stalks (Figure 1G) and none of the spores contained CotC-mRFP (Figure 1H). This indicated that the spores were all derived from the Ax2 cells and that the sporulation defect of RT11da2 is cell-autonomous.

Sequencing of the amplified region that flanked the plasmid insertion site showed that insertion occurred at a *Dpn*II site at nt 7,044 in gene DDB_G0279149, which encodes 1-phosphatidylinositol-3-phosphate 5-kinase (Pip5K3), also known as PIKfyve. To confirm that the observed sporulation phenotype is due to a lesion in *pikfyve*, we deleted a large segment of the *pikfyve* coding region by homologous recombination (Supplementary Figure 1). The *pikfyve*^−^ mutant showed the same fruiting body morphology as clone RT11da2, with enlarged lower stalks and with maturing spores becoming progressively vacuolated. The developmental defects were less severe when cells were plated at a 10-fold lower cell density than is routinely used (Supplementary Figures 2A,B). Mostly normal spores were formed in fruiting bodies developed at the lower cell density (2.5 × 10^5^ cells/cm^2^), but 94% of these spores disintegrated within their walls after 7 days in the spore head. For wild-type, this occurred to 45% of spores (Supplementary Figures 2C,D).

**FIGURE 2:**
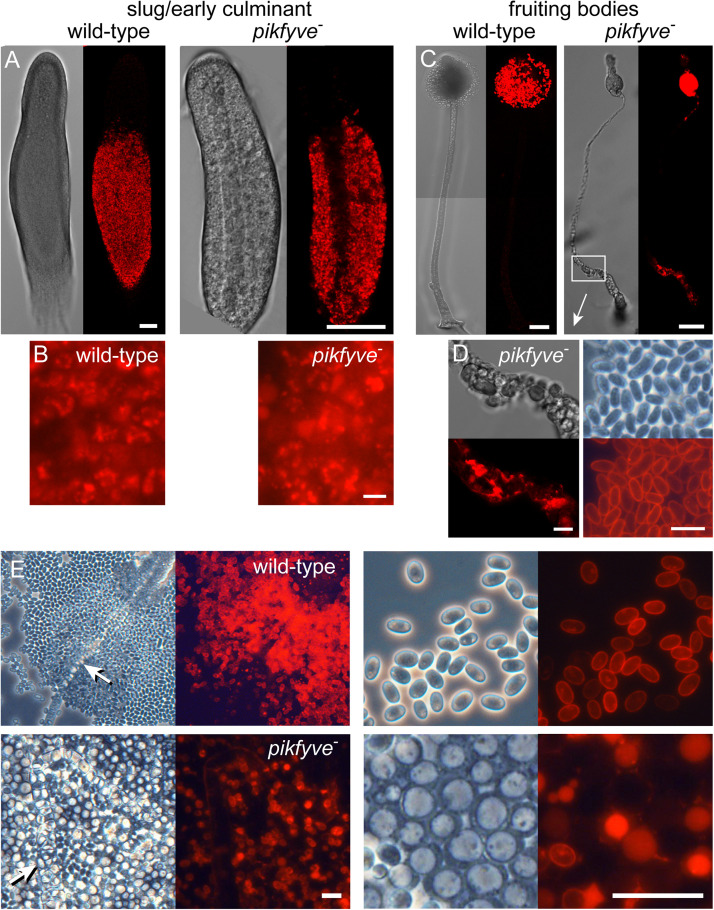
Spore and stalk cell differentiation in the *pikfyve* knockout. **(A–D)** Intact structures. Ax2 and *pikfyve*^−^ cells, transformed with *cotC-RFP*, were developed on non-nutrient agar at 2.5 × 10^5^ cells/cm^2^ into migrating slugs for Ax2 and early culminants for *pikfyve*^−^ (which does not migrate) **(A,B)** and into fruiting bodies **(C,D)** and imaged by confocal microscopy. Bars **(A,C)** 50 μm; **(B,D)** 10 μm. **(E)** Spore heads. Fruiting bodies of Ax2 and *pikfyve*^−^ developed at 2.5 × 10^6^ cells/cm^2^ were transferred to a slide glass and imaged at low and high magnification. Stalks are indicated by arrows. Bars: 20 μm.

We examined the sporulation phenotype further in *pikfyve*^−^ cells, transformed with *cotC-mRFP* (Figure 2). Initially CotC-mRFP was expressed in the posterior prespore region of the sorogens in the same granular pattern as shown by wild-type slugs, indicating that *pikfyve*^−^ normally initiated spore coat synthesis in Golgi-derived prespore vesicles (Figures 2A,B). In *pikfyve*^−^ fruiting bodies, developed at low cell density, stalk and spore formation were almost normal (Figures 2C,D). However, the thick lower stalk consisted of vacuolated cells that also showed CotC-mRFP staining, indicating that they were derived from prespore cells. When developed at high cell density, the *pikfyve*^−^ cells formed some spores with CotC-mRFP positive cell walls, indicating that their prespore vesicles had exocytosed. However, most spores were vacuolated with the CotC-mRFP now present inside the vacuole and not outside on the cell wall (Figure 2E).

### Stalk and Basal Disc Differentiation in *pikfyve^−^*

**FIGURE 3:**
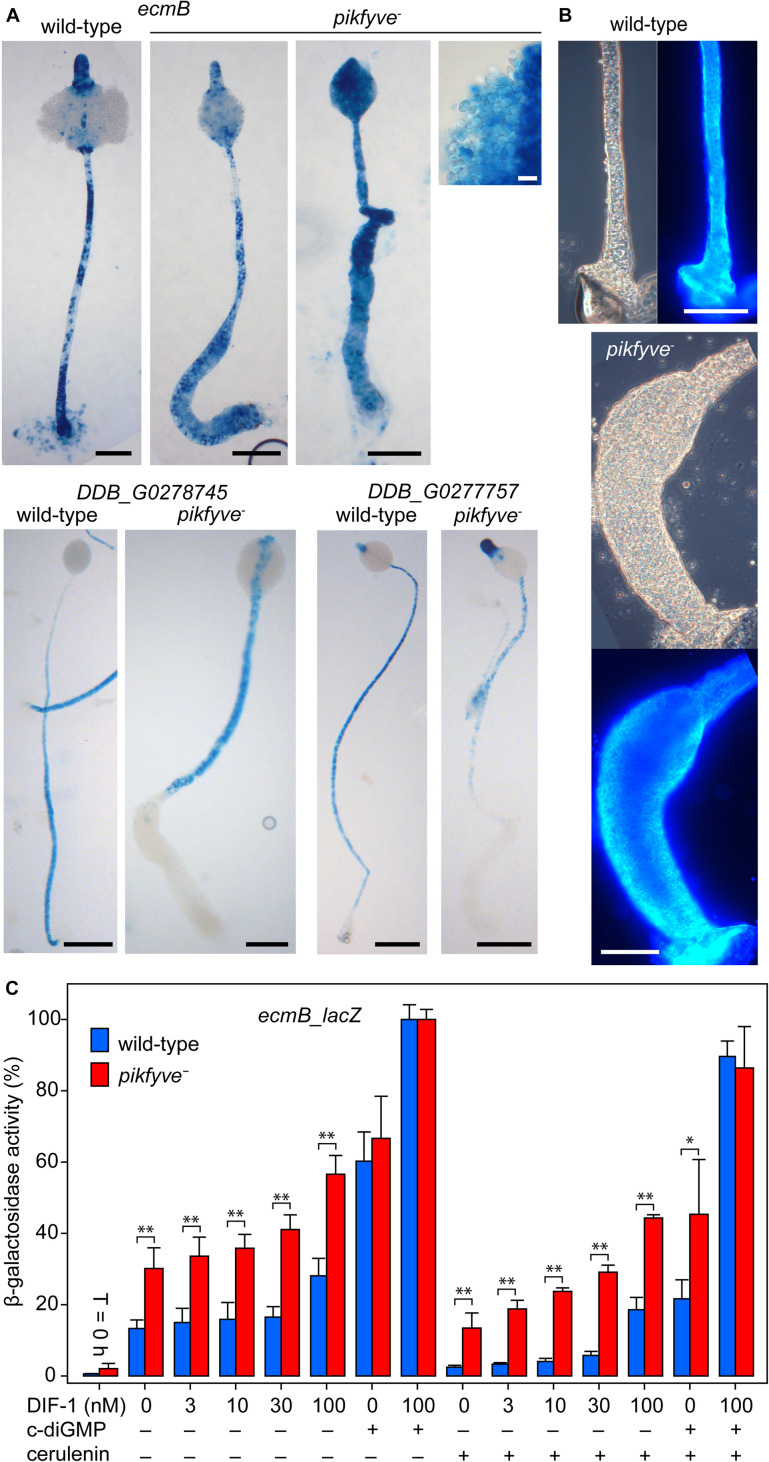
Stalk and basal disc cell differentiation in *pikfyve^−^.* **(A)** Stalk gene expression. Ax2 and *pikfyve*^−^ cells, transformed with *ecmB-lacZ* or *DDB_G0278745-lacZ* gene fusions, were incubated on dialysis membrane supported by non-nutrient agar until fruiting bodies had formed. Structures were fixed and stained with X-gal. Bars: black 100 μm, white 10 μm. **(B)** Calcofluor staining. Wild-type and *pikfyve*^−^ fruiting bodies were lifted onto a slide glass, stained with 0.001% Calcofluor and imaged under phase contrast and epifluorescence. Bars: 100 μm. **(C)** Stalk gene induction. Ax2 and *pikfyve*^−^ cells, transformed with *ecmB-lacZ* were developed into tipped mounds, dissociated in stalk salts (Berks and Kay, 1988) and incubated at 10^6^ cells/ml for 8 h with the indicated concentrations of DIF-1, 3 μM c-di-GMP and/or 100 μM cerulenin and assayed for β-galactosidase activity, using ONPG as substrate. Data are expressed as percentage of β-galactosidase activity obtained in the presence of 100 nM DIF and 3 μM c-di-GMP and represent means and SD of two experiments performed in triplicate. Significant differences between Ax2 and *pikfyve*^−^ are indicated by ^∗^ for *P* < 0.05 and by ^∗∗^ for *P* < 0.001.

Extreme vacuolization is widely reported to accompany deletion of PIKfyve function (Gary et al., 1998; Nicot et al., 2006; Whitley et al., 2009; Buckley et al., 2019). However, in *D. discoideum* extreme vacuolization is also the hallmark of the stalk cells and of the cells that form a basal disc to support the stalk. Both stalk and basal disc cells express stalk-specific genes and are encapsulated by a cellulosic wall that is distinct from the spore wall. To assess the identity of the stalk-like vacuolated cells in the *pikfyve*^−^ mutant, we examined the expression pattern of *ecmB*, a gene that is expressed in stalk and basal disc cells (Jermyn and Williams, 1991), and of *DDB_G0278745 DDB_G0277757* that are only expressed in the stalk (Kin et al., 2018). *Pikfyve*^−^ and its Ax2 parent strain were transformed with fusion constructs of the promoters of these genes and the *LacZ* reporter. Figure 3A shows that *ecmB* is expressed in both the stalk and lower enlarged region of the stalk of *pikfyve*^−^ fruiting bodies, while *DDB_G0278745* and *DDB_G0277757* are only expressed in the stalk. This suggests that the widened lower stalk consists of basal disc cells. Staining with the cellulose dye Calcofluor showed that the cells in the widened lower stalks of *pikfyve*^−^ fruiting bodies are surrounded by a cellulose wall and not merely vacuolated (Figure 3B). Because such cells previously expressed *cotC* (Figure 2C), they must have transdifferentiated from prespore cells into basal disc cells.

Phenotypically, the stalk cells only differ from the basal disc cells by being communally enclosed in the cellulose stalk tube. Mutants that cannot synthesize the secreted polyketide DIF-1 do not form the basal disc, but also have somewhat weaker stalks, suggesting that DIF-1 also contributes to stalk cell differentiation (Saito et al., 2008). 3′,5′-cyclic diguanylic acid (c-di-GMP), synthesized by the prestalk-specific enzyme diguanylate cyclase (dgcA) is essential for stalk formation (Chen and Schaap, 2012). C-di-GMP acts by hyperactivating adenylate cyclase A (acaA), which is highly expressed at the slug tip, where stalk formation initiates. The increased cAMP levels subsequently activate PKA to induces stalk gene expression and stalk cell maturation (Chen et al., 2017). Within the intact structure, c-di-GMP acts specifically on tip cells, because both dgcA and acaA are specifically expressed in prestalk and tip cells, respectively (Verkerke-van Wijk et al., 2001; Chen and Schaap, 2012). In cell suspension, high concentrations of c-di-GMP also induce expression of cup and spore genes, which are also under positive control of PKA. Unlike DIF-1, which diverts prespore cells into the stalk-like basal disc cells, c-di-GMP does therefore not impose initial cell fate. Since DIF-less mutants still form the stalk (Saito et al., 2008), the actual signal that induces stalk precursors is unknown. We tested whether the excessive differentiation of stalk-like cells in *pikfyve*^−^ was due to increased responsiveness to either DIF-1 or c-di-GMP. In a semi-quantitative monolayer assay, where cells are starved with 1 mM cAMP to render them competent to DIF-1 (Berks and Kay, 1988), both DIF and c-di-GMP appeared to induce stalk-like cell vacuolization more effectively in *pikfyve*^−^ than in Ax2 cells (Supplementary Figure 3). In an *ecmB-lacZ* gene induction assay, starting with cells from dissociated tipped mounds, *ecmB* induction by DIF-1 was also higher in *pikfyve*^−^ than in Ax2 cells and induction even seemed to occur in the absence of DIF-1 (Figure 3C). c-di-GMP induced *ecmB* expression equally in Ax2 and *pikfyve*^−^ and yielded an even higher level of induction when combined with DIF-1. To assess whether *ecmB* induction without added DIF-1 was due to endogenously produced DIF-1, we included the polyketide synthase inhibitor cerulenin (Sato et al., 2016) in the incubation medium. This only partially reduced unstimulated *ecmB* expression in *pikfyve*^−^ and left DIF-stimulated expression still well above that of Ax2. c-di-GMP activated *ecmB* expression was also higher in *pikfyve*^−^ than Ax2. When comparing the responses to different concentrations of DIF-1 between *pikfyve*^−^ and Ax2, the *pikfyve*^−^ cells do not appear to be more sensitive to DIF-1 (i.e., to respond to lower DIF-1 concentrations). Instead the effects of DIF-1 and the *pikfyve* lesion seem to be additive.

To investigate whether the *pikfyve* lesion acts independently of DIF-1, we examined whether inhibition of DIF-1 synthesis by cerulenin ameliorates the developmental defect of *pikfyve*^−^ cells. In Ax2, cerulenin caused formation of fruiting bodies with thin and sagging spore heads, as also described for DIF-less mutants (Saito et al., 2008). Cerulenin had severe adverse effects on *pikfyve*^−^ cells and prevented most of the aggregated cells from being incorporated in fruiting bodies. These cells, and those occupying the lower stalk region remained mostly amoeboid (Supplementary Figure 4). While cerulenin therefore did not ameliorate the *pikfyve*^−^ culmination defect, it did counteract excessive stalk-like cell differentiation, suggesting some dependence of its phenotype on DIF-1 or other polyketides.

We also tested the effects of DIF-1 and cerulenin on the extensive vacuolization of early starving *pikfyve*^−^ cells that was observed by Buckley and coworkers (Buckley et al., 2019). Supplementary Figure 5 shows that up to 200 nM DIF-1 does not markedly increase vacuolization of Ax2 cells after 8 h of incubation, while neither DIF-1 nor cerulenin have a notable effect on the extensive vacuolization that occurs in *pikfyve*^−^ cells. Obviously, this early effect of the *pikfyve*^−^ lesion is independent of DIF-1. As reported by Buckley et al., we also noted that during longer starvation *pikfyve*^−^ cells lose their hypervacuolated state.

### Autophagy and Prespore Gene Induction in *pikfyve^−^*

Transdifferentiation of prespore into basal disc cells was also observed in *knkA*^−^ and *bcas3*^−^ mutants, which are partially defective in early autophagosome formation (Yamada and Schaap, 2020). More severe autophagy-deficient mutants with lesions in the autophagy genes *atg5*, *atg7* or *atg9* almost completely lacked prespore and spore differentiation and massively overproduced stalk-like cells (Yamada and Schaap, 2019). Prespore differentiation is induced in sorogens by secreted cAMP acting on cAMP receptors (Schaap and Van Driel, 1985; Wang et al., 1988). The *atg5*^−^, *atg7^−^*, and *atg9*^−^ mutants completely lacked cAMP induction of prespore differentiation and this was partially the case for the *knkA*^−^- and *bcas3*^−^ mutants.

**FIGURE 4:**
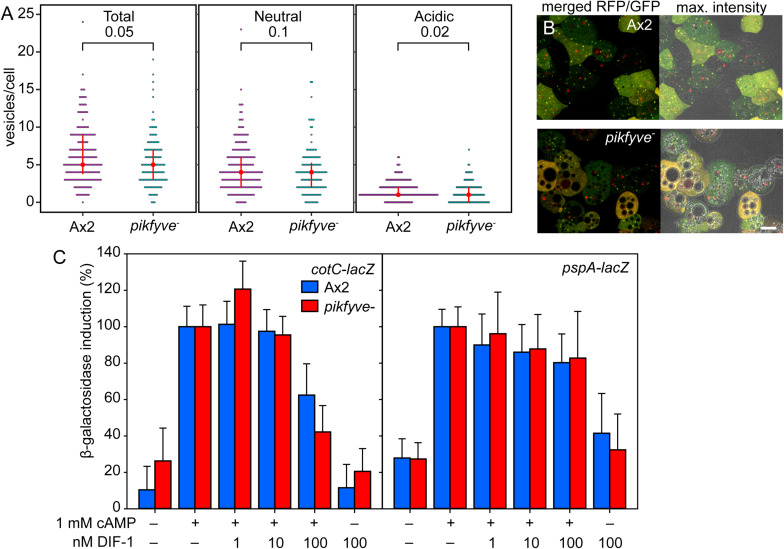
Autophagy and prespore gene induction in *pikfyve^−^.* **(A,B)** Autophagy. Ax2 and *pikfyve*^−^ cells transformed with RFP-GFP-Atg8 were starved until aggregates were forming and imaged by confocal microscopy (Yamada and Schaap, 2020). Z series of images in 0.5 μm steps were captured and the maximum intensity projection of these images, as shown in **(B)**, right panel, was used to quantify Atg8 vesicles. Numbers of vesicles were counted in each cell that show either both RFP and GFP fluorescence (Neutral), or only RFP fluorescence (Acidic), and the sum of neutral and acidic vesicles (Total) was calculated. Red circles and red bars represent the median and quartile of each data set, respectively. *P*-values of a Wilcoxon rank-sum test are shown above brackets. 188 and 120 cells were counted for Ax2 and *pikfyve*^−^, respectively. **(B)** Left panel: merged RFP and GFP optical section. Bar: 10 μm. **(C)** Prespore gene induction. Ax2 and *pikfyve*^−^ cells, each transformed with *cotC* or *pspA* promoter-*lacZ* fusion constructs, were developed into loose aggregates and incubated for 4 h at 10^6^ cells/ml for *cotC-lacZ* and 8 h at 2 × 10^6^ cells/ml for *pspA-lacZ* with the indicated variables in microtiterplate wells. Cells were assayed for β-galactosidase activity, using CPRG as substrate. Means and SD of three experiments performed in triplicate are presented.

We investigated whether the *pikfyve*^−^ mutant is also defective in autophagy and/or cAMP induction of prespore differentiation. To measure autophagy, Ax2 and *pikfyve*^−^ cells were transformed with pRFP-GFP-Atg8, which labels early autophagosomes of neutral pH yellow, due to combined RFP and GFP fluorescence, and mature autolysosomes red, due to quenching of GFP fluorescence by low pH (Kimura et al., 2007; Domínguez-Martín et al., 2017). Cells were starved until aggregates were forming and imaged by confocal microscopy (Figures 4A,B). Although the extensive vacuolization of the *pikfyve*^−^ cells (Figure 4B) may have affected vesicle counting somewhat, the numbers per cell of neutral and acidic Atg8 positive vesicles were not markedly different between Ax2 and *pikfyve^−^.* This indicates that early autophagy occurred normally in *pikfyve^−^.*

To measure prespore gene induction, Ax2 and *pikfyve*^−^ cells were transformed with gene fusions of *LacZ* and promoters of the prespore genes *pspA* (Dingermann et al., 1989) and *cotC* (Fosnaugh and Loomis, 1989). Dissociated loose aggregates were incubated with 1 mM cAMP and increasing concentrations of DIF-1, which apart from inducing basal disc differentiation also inhibits prespore differentiation (Wang et al., 1986; Morris et al., 1987; Saito et al., 2008). Figure 4C shows that cAMP is equally effective in inducing *cotC-lacZ* and *pspA-lacZ* expression in wild-type cells and in *pikfyve*^−^ cells. This indicates that, unlike loss of autophagy genes, loss of PIKfyve does not prevent cAMP induction of prespore gene expression. There was also no marked difference in DIF-1 inhibition of prespore gene induction between wild-type and *pikfyve*^−^ cells. Combined, the normal autophagosome formation in *pikfyve*^−^ and its normal cAMP induction of prespore gene expression suggests that the transdifferentiation of prespore into basal disc cells in *pikfyve*^−^ must have another cause than defective early autophagy.

### Roles for Fig4 and Vac14 in *Dictyostelium* Development

**FIGURE 5:**
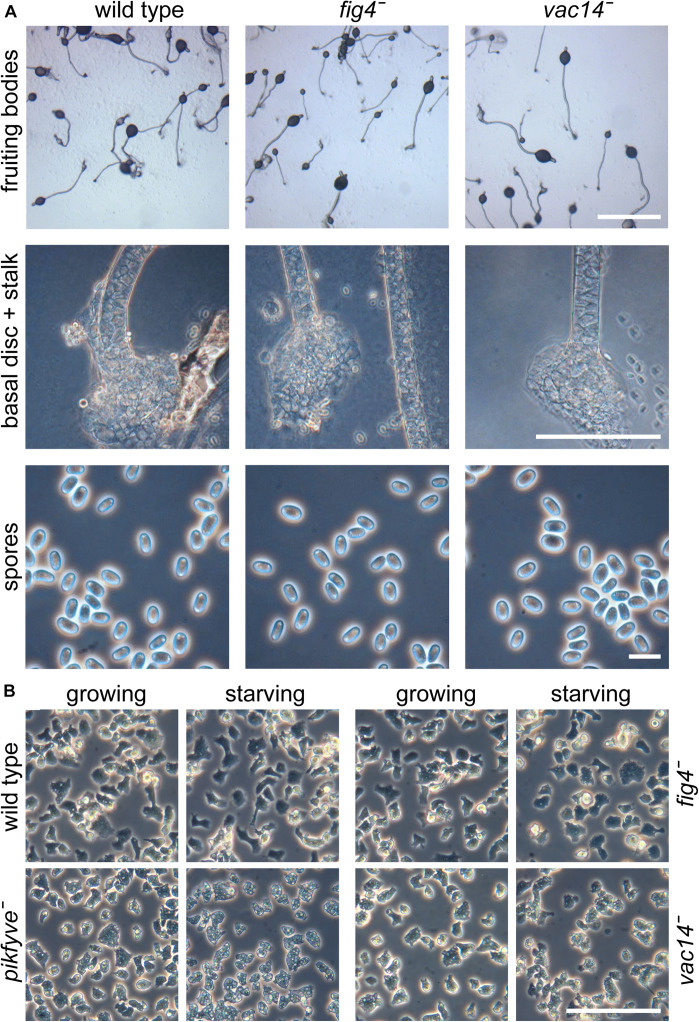
Phenotypes of *fig4*^−^ and *vac14*^−^ mutants. **(A)** Multicellular structures. Fig4 (DDB_G0281427) and *vac14* (DDB_G0289233) were knocked out by homologous recombination (Supplementary Figure 7). Knock-out cells and the Ax2 parent were plated for development at 2.5 × 10^6^ cells/cm^2^ on non-nutrient agar and developed into fruiting bodies. Intact structures, lower stalks and spores were photographed under transmitted light and phase contrast. Bar at top: 1 mm, center: 100 μm, bottom: 10 μm. **(B)** Amoebas. Ax2, *pikfyve*^−^, fig4*^−^*, and *vac14*^−^ cells were deposited at 2.5 × 10^5^ cells/cm^2^ in glass-bottomed petri-dishes in either HL5 axenic medium (growing) or 20 mM K-phosphate, pH 6.2 (starving) and photographed under phase contrast after 4 h at 22°C. Bar: 100 μm.

In yeast and other organisms, PIKfyve acts in a complex with the scaffolding protein Vac14, the 5-phosphatase Fig4 and the autophagy protein Atg18 (Ho et al., 2012). Fig4 acts antagonistically to PIKfyve by hydrolyzing the 5-phosphate of PtdIns(3,5)P2, but is like Vac14 also required for PIKfyve activity (Dove et al., 2002; Duex et al., 2006). Atg18 is a downstream effector of PIKfyve, with its membrane scission activity being activated by PtdIns(3,5)P2 (Gopaldass et al., 2017). If loss of PIKfyve promotes basal disc differentiation, we would expect loss of Fig4 to inhibit or decrease it, and loss of Vac14 to replicate the *pikfyve*^−^ phenotype. Atg18 also functions in early autophagosome assembly and other proteins such as Atg5, Atg7 and Atg9, which also participate in this process, where shown to be required for early prespore differentiation (Yamada and Schaap, 2019). Deletion of *atg18* is therefore unlikely to be informative about the downstream process of spore maturation. BLASTp queries and phylogenetic inference show that orthologs of yeast and mammalian *pikfyve*, fig4 and *vac14* are present as single copy genes throughout Dictyostelia and other Amoebozoa (Supplementary Figure 6). We deleted fig4 and *vac14* by homologous recombination in *D. discoideum* (Supplementary Figure 7). Both the fig4*^−^* and *vac14*^−^ cells formed well-proportioned fruiting bodies with normal spores, stalks and basal discs (Figure 5A). Axenically growing and particularly early starving *pikfyve*^−^ cells also show a hypervacuolated phenotype (Buckley et al., 2019). We found that also *vac14*^−^ cells showed hypervacuolation, but not to the same extent as *pikfyve*^−^ (Figure 5B). The phenotype of growing or starving fig4*^−^* cells was similar to that of the parent Ax2. These data suggest that at least *pikfyve*^−^ and *vac14*^−^ likely interact with each other in *D. discoideum* as they do in other organisms. However, both the reduced vacuolization of *vac14*^−^ cells and their normal multicellular development indicate that PIKfyve activity is not completely dependent on Vac14.

## Discussion

### PIKfyve Is Required for Viable Spore Formation in *Dictyostelium*

*PIKfyve* was identified as the defective gene in a screen for mutants that lack normal spore differentiation. The initial selection criterium was lack of expression of the RFP-labeled spore coat protein *cotC* in fruiting bodies, but both the initial insertional mutant and a *pikfyve* knock-out created by homologous recombination also displayed other morphological abnormalities, notably a very thick lower stalk. The developmental defects were to some extent conditional. When developed at low cell density, only the lower stalk was expanded and initial spore differentiation seemed normal (Figures 1, 2 and Supplementary Figure 2).

A *D. discoideum pikfyve^−^* mutant studied previously was reported to show a reduced growth rate on both bacteria and in axenic medium, the latter accompanied by enlarged macropinocytic vacuoles. These large vacuoles persisted for several hours after starvation, but disappeared as amoebas entered into aggregation (Buckley et al., 2019). Further fruiting body formation on filters was reported to be normal, but this may have been due to the specific developmental condition being used. The work focusses on the early hypervacuolization defect, which is shown to be accompanied by a lack of V-ATPase delivery to and acidification of the phagosomes.

### Prespore Cells Transdifferentiate Into Basal Disc Cells in *pikfyve^−^*

*pikfyve*^−^ cells, developed both at high and low cell density, initially showed normal prespore differentiation in the posterior three-quarters of the emerging sorogen. The formation of the primary stalk also occurred normally. However, at low cell density, a proportion of the prespore cells at the base of the structure then transdifferentiated into stalk-like cells with a single large vacuole and cellulose wall. At high cell density, the greater majority of prespore cells transdifferentiated into stalk-like cells. We surmise that this cell density effect is due to increased accumulation of secreted signals or catabolites at higher cell density that exacerbate the effect of the *pikfyve* lesion. The stalk-like cells expressed the stalk gene *ecmB* that is expressed both in the primary stalk and the basal disc, but not gene *DDB_G0278745* that is only expressed in the stalk, suggesting that the prespore cells had transdifferentiated into basal disc cells. When developed mixed with wild-type cells, none of the *pikfyve-* cells participated in the spore population of the chimeric fruiting bodies (Figure 1H), indicating that the *pikfyve* lesion imparts a strongly cell-autonomous bias against spore differentiation.

### Loss of *pikfyve^−^* Does Not Affect Autophagy or Prespore Gene Induction

The *pikfyve*^−^ developmental phenotype is similar, but less severe than that of two other sporulation mutants that were identified in the same mutant screen and that proved to be defective in the known autophagy gene *atg7* and in a novel autophagy gene *knkA*. The *atg7*^−^ and also *atg5*^−^ and *atg9*^−^ mutants showed greatly reduced prespore differentiation, loss of slug migration and transdifferentiation of prespore cells into basal disc cells. All three mutants specifically lacked cAMP induction of prespore gene expression (Yamada and Schaap, 2019). Autophagy was not fully lost in *knkA*^−^ and neither was slug migration, while cAMP induction of prespore gene expression was about 50% reduced (Yamada and Schaap, 2020).

However, *pikfyve*^−^ cells normally cycled the autophagy marker RFP-GFP-Atg8 from neutral to acidic compartments and showed normal cAMP induction of prespore differentiation (Figure 4), indicating that the *pikfyve* lesion affects another aspect of the developmental program. In *pikfyve*^−^ cells, both basal and DIF-1 induced expression of the stalk/basal disc gene *ecmB* were 2–3-fold higher than in wild-type cells. Because DIF-1 is essential for basal disc rather than stalk differentiation (Saito et al., 2008) and the basal disc is particularly enlarged in *pikfyve^−^*, it therefore appears that the sporulation defect of *pikfyve*^−^ is due to enhanced basal disc gene expression, rather than defective induction of prespore gene expression, as is the case for the autophagy mutants.

### Is *Dicytostelium* Development Regulated by Its Endosomal System?

Lesions in *pikfyve* also result in a hypervacuolated phenotype in animals and yeast. This is caused by the lack of PtdIns3,5(P)2 formation in the lysosomal and yeast vacuolar membranes, which is required to activate the membrane scission activity of Atg18 (Gopaldass et al., 2017) as part of normal recycling of these organelles. The reported size increase of phagosomes of *Dictyostelium pikfyve*^−^ mutants (Buckley et al., 2019) likely has the same cause. However, the effect of the *pikfyve*^−^ lesion in causing the transdifferentiation of prespore cells and spores into stalk cells is more enigmatic, since it not only involves hypervacuolization, but also the activation of stalk-like gene expression and stalk wall biosynthesis. The same is true for lesions in *Dictyostelium* autophagy genes, which have a specific inhibitory effect on induction of prespore gene expression and ultimately also lead to transdifferentiation of prespore cells into basal disc cells (Yamada and Schaap, 2019, 2020). The stage in which the genes act on endosome processing mirrors the stage in which their disruption acts on sporulation, with deletion of *atg5, 7* and *9*, which are all involved in early autophagosome assembly, perturbing the earliest step in spore differentiation, and deletion of *pikfyve*, which acts late in autolysosome processing, acting late in spore maturation. While this may yet prove to be coincidental, it remains of great interest to test involvement of more endosomal genes in sporulation and *vice versa*, and to elucidate how endosomal genes can have such direct effects on cell type specific gene expression.

## Data Availability Statement

The original contributions presented in the study are included in the article/Supplementary Material, further inquiries can be directed to the corresponding author/s.

## Author Contributions

YY and PS designed the study and wrote the manuscript. YY, QD, and GF performed the experiments. TK and PS supervised the experimentation. All authors contributed to the article and approved the submitted version.

## Conflict of Interest

The authors declare that the research was conducted in the absence of any commercial or financial relationships that could be construed as a potential conflict of interest.

## Publisher’s Note

All claims expressed in this article are solely those of the authors and do not necessarily represent those of their affiliated organizations, or those of the publisher, the editors and the reviewers. Any product that may be evaluated in this article, or claim that may be made by its manufacturer, is not guaranteed or endorsed by the publisher.
