# Supplementary material for: Loss of PIKfyve Causes Transdifferentiation of Dictyostelium Spores Into Basal Disc Cells
Source: Front Cell Dev Biol. 2021 Aug 19;9:692473. doi: 10.3389/fcell.2021.692473 (PMC8417116; doi:10.3389/fcell.2021.692473)
Supplement: Supplementary file 1 [file Presentation_1.PDF]

SUPPLEMENTARY MATERIAL

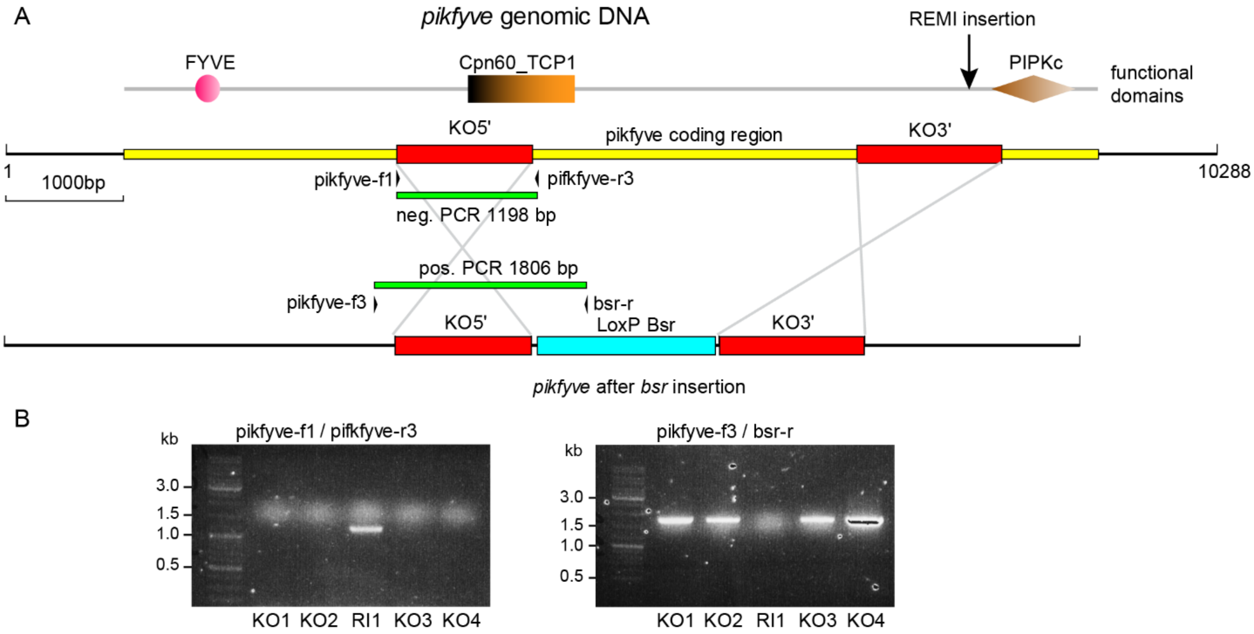

Figure S1. Schematic of the pPIKfyveKO construct and knock-out diagnosis
A. *KO construct*. Genomic region and functional domain architecture of the *pikfyve* gene and a schematic of the knock-out construct. Arrowheads show the position of the primers used for knock-out (KO) diagnosis.
B. *Diagnosis*. Primer pair pikfyve-f1/pikfyve-r3 (Table S1) amplifies a 1.2 kb fragment from the random integrant (RI), but not the KO gDNAs, whereas primer pair pikfyve-f3/bsr-r amplifies a 1.8 kb fragment from KO gDNAs only.

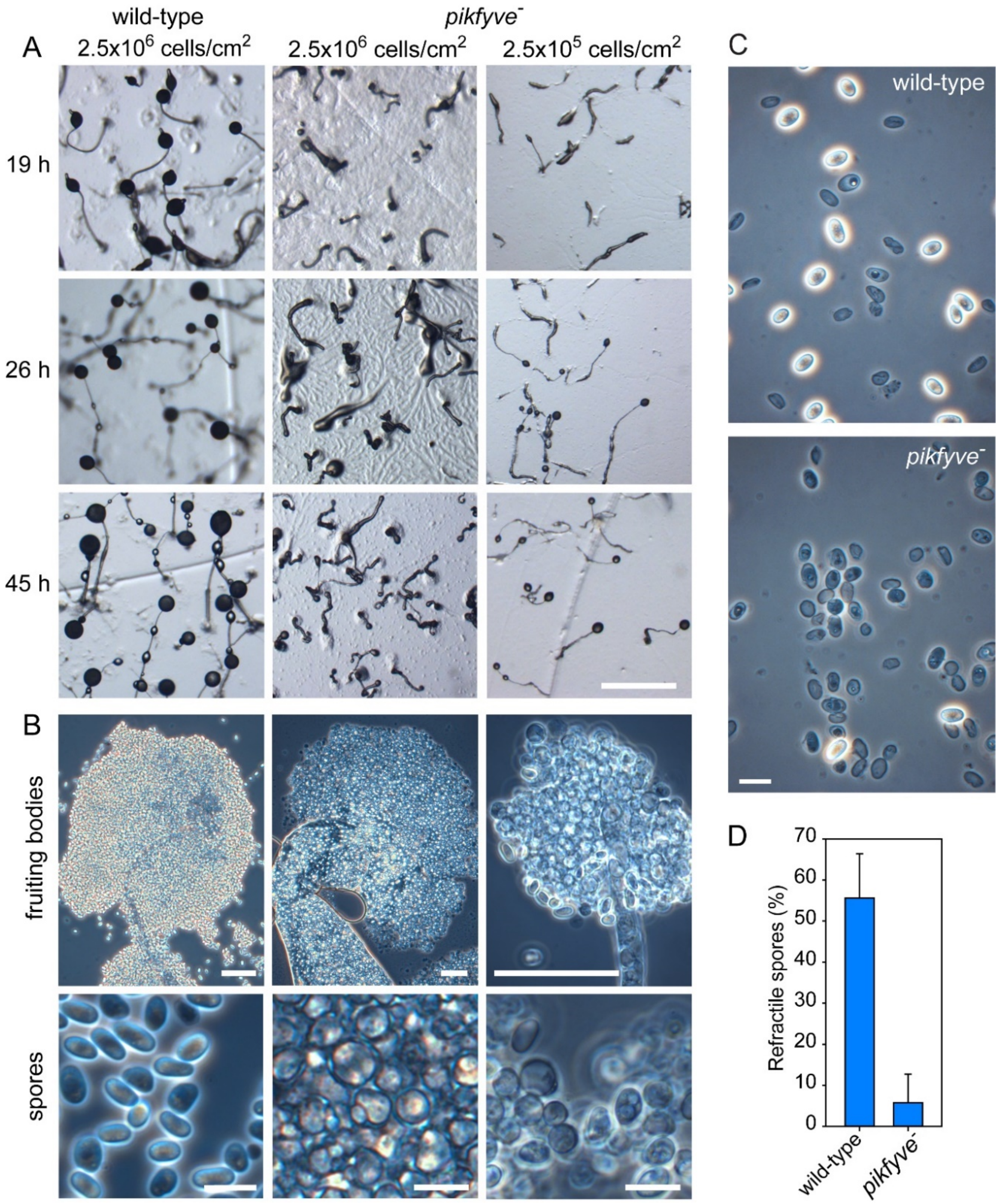

Figure S2. Effect of cell density on *pikfyve*⁻ development

A. Wild-type Ax2 and *pikfyve*⁻ cells were plated on non-nutrient agar at $2.5x10^6$ and $2.5x10^5$ cells/cm^2 and photographed after the indicated hours of incubation at 22ºC. Bar: 1 mm

B. Mature fruiting bodies were squashed under coverslips and spore heads were photographed under low (top) and high (bottom) magnification. Bars top: 50 µm, bottom: 10 µm.

C/D. Spores harvested from 1-week old fruiting bodies, developed at $2.5x10^5$ cells/cm^2, were imaged (Bar: 10 µm) and the percentage of refractile viable spores was determined (D). Means and SD are shown of 843 spores measured over 3 samples for wild-type and 590 spores measured over 5 samples for *pikfyve*⁻.

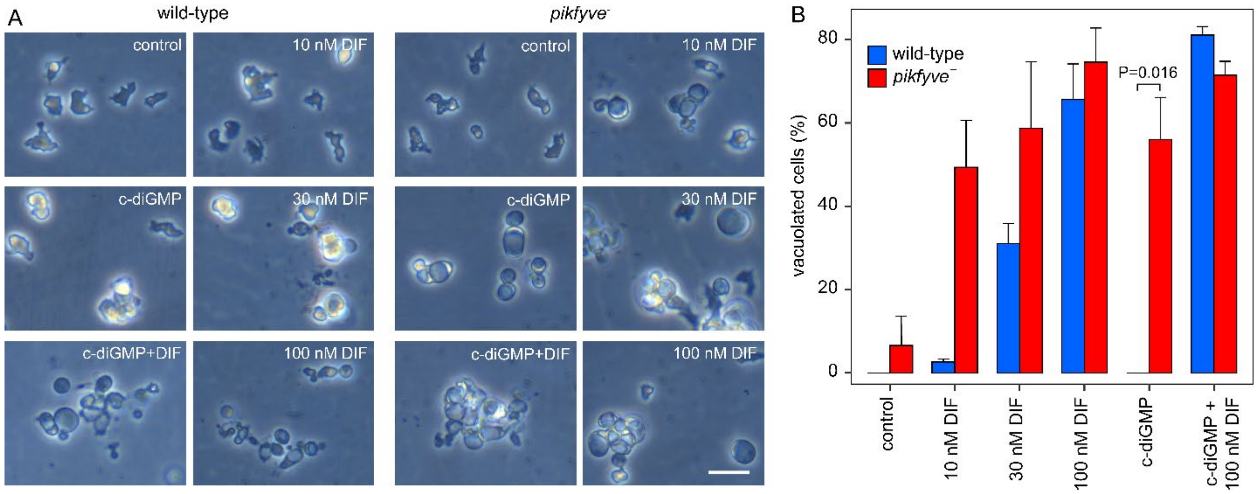

Figure S3. Stalk cell differentiation in monolayers

Ax2 and *pikfyve*⁻ cells were pre-incubated for 6 h with 1 mM cAMP. After removal of cAMP, cells were incubated at 2 x 10^5 cells/ml with the indicated concentration of DIF-1 and/or or 3 mM c-diGMP. Control cells received the DIF-1 solvent, 0.1% ethanol. A. Images taken at 23 hours of incubation, Bar: 20 µm. B. After 18 h at 22ºC, the fraction of vacuolated cells was determined. Means and SD of 2 or 3 experiments are shown, with 39 to 173 cells were counted in each sample. Due to formation of cell clumps, exact quantitation was problematic and a significant difference between Ax2 and *pikfyve*⁻ cells was only observed for cells treated with 3 µM c-di-GMP only.

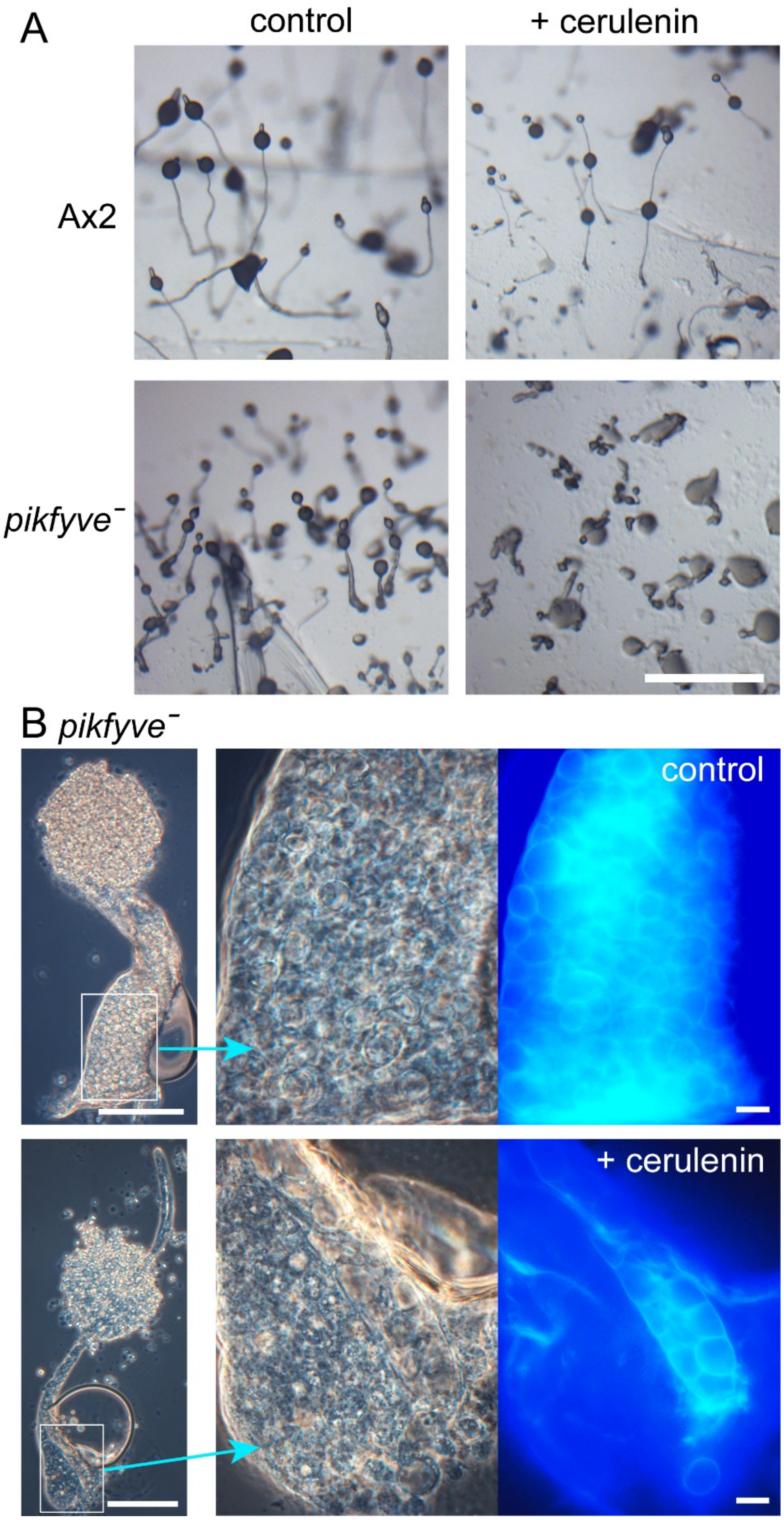

Figure S4. Effect of cerulenin on *pikfyve*⁻ development

A. Ax2 and *pikfyve*⁻ cells were developed on non-nutrient agar containing 0.01% ethanol (control) or 0.5 mM cerulenin and terminal fruiting structures were photographed after 24 h. Bar: 1 mm.

B. After 48 h of development, *pikfyve*⁻ structures were lifted onto a slide glass, which separated them from the loose undifferentiated cell mass, stained with 0.001% Calcofluor and imaged under phase contrast and UV. Bars left image: 100 μm, right images: 10 μm.

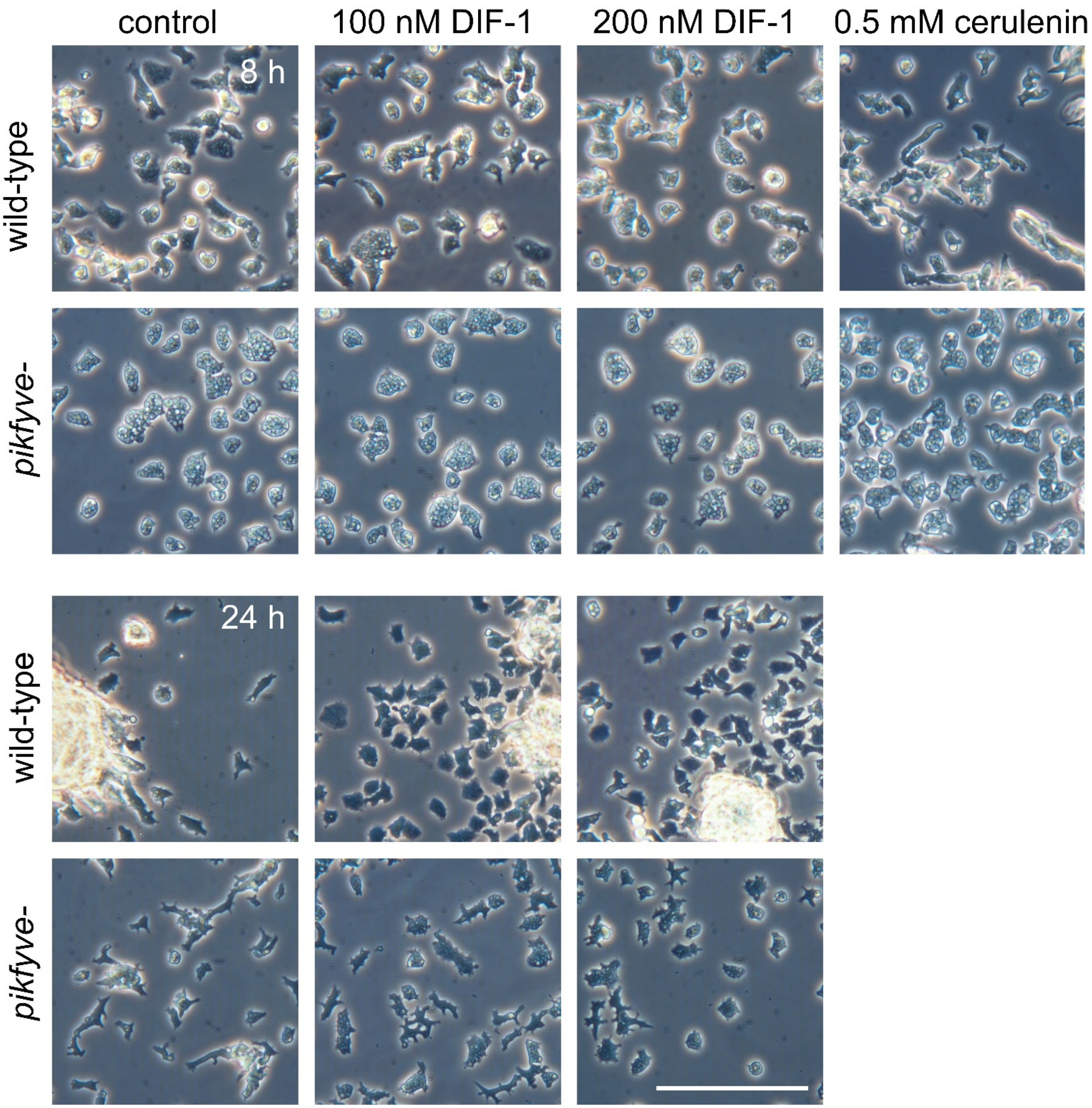

Figure S5. Effects of DIF-1 and cerulenin on early vacuolization of *pikfyve*⁻ and wild type cells
Wild-type Ax2 and *pikfyve*⁻ cells were harvested from growth medium, resuspended in stalk salts and deposited at 2.5 x 10^5 cells/cm^2 in glass-bottomed petri-dishes with the indicated concentrations of DIF-1 and cerulenin. Cells were photographed under phase contrast after 8 h and 24 h at 22ºC. Bar: 100 μm.

Note that the *pikfyve*$^{-}$ cells are vacuolated after 8 h of starvation but have lost extensive vacuolization at 24 h. Somewhat later than wild-type, *pikfyve*$^{-}$ amoebas then also aggregate under submerged conditions.

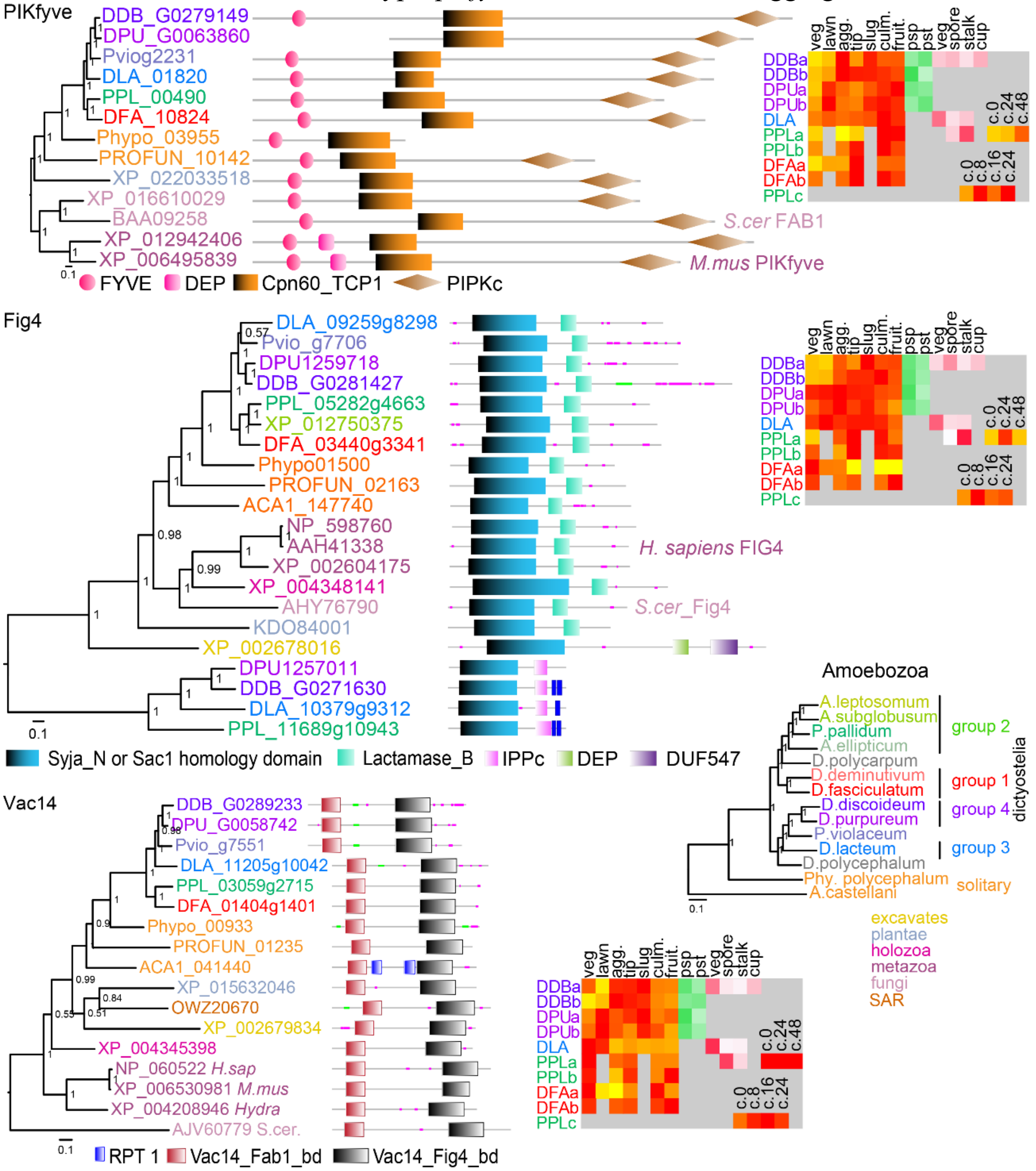

Figure S6. Annotated phylogenies of Pikfyve and its interactors Fig4 and Vac14

Pikfyve, Fig4 and Vac14 proteins were retrieved from Amoebozoan genomes by local BlastP search with yeast or human sequences, while close homologs of the *D. discoideum* proteins in other eukaryote divisions were identified by BlastP search of NCBI within individual divisions. Protein sequences were aligned using Clustal Omega (Sievers and Higgins, 2014) and phylogenetic trees were inferred using MrBayes 3.1 (Ronquist and Huelsenbeck, 2003) . The tree were annotated with the domain architecture of the proteins as determined by SMART (Schultz et al., 1998) and for the dictyostelid genes with heat maps of the developmental expression profiles, relative expression in prestalk and prespore cells of slugs, and relative expression in growing cells and the mature stalk, spore and cup cells of fruiting bodies

(Gloeckner et al., 2016;Kin et al., 2018;Parikh et al., 2010). For *P. pallidum* regulation during development to cysts is also shown (time points in hours, preceded by c.).

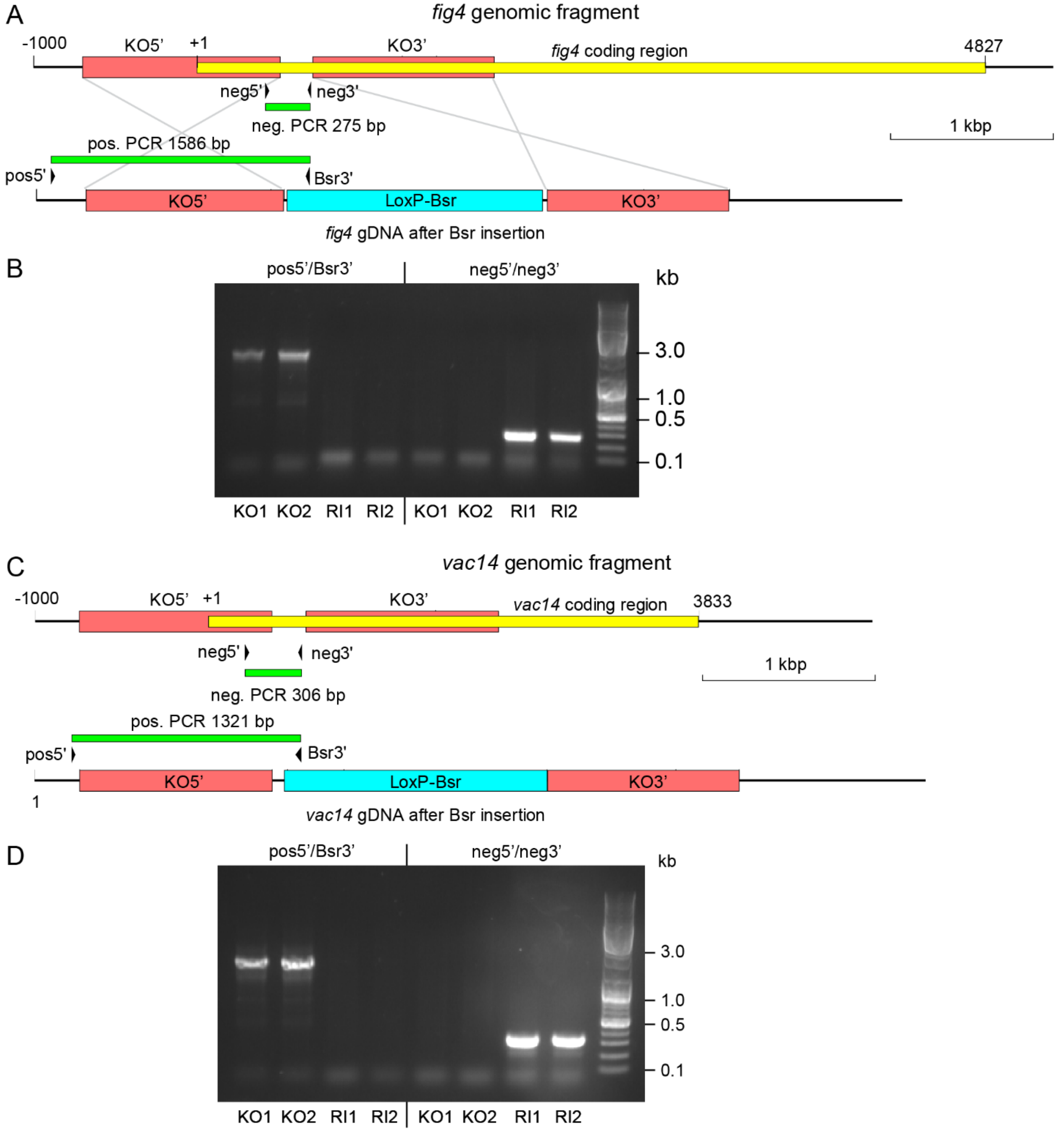

Figure S7. Knock-out schematics and diagnosis for *fig4* and *vac14* knock-outs
A/C. *KO construct*. Genomic regions the *fig4* (A) and *vac14* (C) genes and schematics of the knock-out construct. Arrowheads show the position of the primers used for knock-out (KO) diagnosis.
B/D. *Diagnosis*. Primer pairs neg5'/neg3' (Table S1) amplify a 275 and 306 bp fragment from the random integrant (RI), but not the KO gDNAs of *fig4* and *vac14*, respectively. Primer pairs pos5'/bsr3' amplify a 1.6 kb fragment from *fig4* KO gDNAs, and a 1.3 kb fragment from *vac14* KO gDNAs.

Table S1 Oligonucleotides used in this work

Name	Sequence
pikfyve-f1	GTCGACCACCAAATTCAGCAAGAGG
pikfyve-f2	CTGCAGTCCAACCATGCCTTATCTC
pikfyve-f3	GACCCAAGACATTCAATGCC
pikfyve-r2	GGATCCATGATCCACTCTTACCACC
pikfyve-r3	TGAGCATGTCTTGATACAGTC
bsr-r	GCCGCTCCCACATGATG
Fig4 I5’	GATGGTACCTGTCATCTTTGATGACATTG
Fig4 I3’	GATGTCGACTCTTGTTAATCCCTCTCT
Fig4 II5’	GATCTGCAGGTTTATTCCTCAGCCTAGA
Fig4 II3’	ACTAGTGCTGCATTTGTTCTATCC
Fig4Neg5’	GATATCAGAGGATCCAACCG
Fig4Neg3’	TTCATCGGCAAATTCTGGTG
Fig4Pos5’	GTCAGCAGGATCATCTCTTA
Bsr3’	GATTTGATGGGATTAATTAATTTGTAATC
Vac14I5’	GATGGTACCATCCTCTACATCTTCTTCAA
Vac14I3’	GATGTCGACTGGTGGTACAATCTCCTGA
Vac14II5’	GATCATATGCAGAATCTCCAACTTTTGAC
Vac14II3’	GATGGATCCATTACCATCTTGATTACC
Vac14neg5’	GGTTTAGCTTCTGTTGCAATTG
Vac14neg3’	ACACCACCTTTTACTTGAGG
Vac14pos3’	CATCCTCTTCTGCATCCTCT

SUPPLEMENTAL REFERENCES

Gloeckner, G., Lawal, H.M., Felder, M., Singh, R., Singer, G., Weijer, C.J., and Schaap, P. (2016). The multicellularity genes of dictyostelid social amoebas. *Nat Commun* 7**,** 12085.

Kin, K., Forbes, G., Cassidy, A., and Schaap, P. (2018). Cell-type specific RNA-Seq reveals novel roles and regulatory programs for terminally differentiated Dictyostelium cells. *BMC Genomics* 19**,** 764.

Parikh, A., Miranda, E.R., Katoh-Kurasawa, M., Fuller, D., Rot, G., Zagar, L., Curk, T., Sucgang, R., Chen, R., Zupan, B., Loomis, W.F., Kuspa, A., and Shaulsky, G. (2010). Conserved developmental transcriptomes in evolutionarily divergent species. *Genome Biol* 11**,** R35.

Ronquist, F., and Huelsenbeck, J.P. (2003). MrBayes 3: Bayesian phylogenetic inference under mixed models. *Bioinformatics* 19**,** 1572-1574.

Schultz, J., Milpetz, F., Bork, P., and Ponting, C.P. (1998). SMART, a simple modular architecture research tool: identification of signaling domains. *Proc. Natl. Acad. Sci. USA* 95**,** 5857-5864.

Sievers, F., and Higgins, D.G. (2014). Clustal omega, accurate alignment of very large numbers of sequences. *Methods in molecular biology* 1079**,** 105-116.
